# Decoupling the chemical and physical origins of the seed spectral manifold in sorghum

**DOI:** 10.1038/s41540-026-00728-w

**Published:** 2026-05-11

**Authors:** Insuck Baek, Seunghyun Lim, Lalit M. Kandpal, Chansong Hwang, Moon S. Kim, Sunchung Park, Louis K. Prom, Lyndel W. Meinhardt, Clint Magill, Ezekiel Ahn

**Affiliations:** 1 Environmental Microbial and Food Safety Laboratory, Agricultural Research Service, United States Department of Agriculture, Beltsville, MD USA; 2 Sustainable Perennial Crops Laboratory, Agricultural Research Service, United States Department of Agriculture, Beltsville, MD USA; 3 Insect Control and Cotton Disease Research, Agricultural Research Service, Southern Plains Agricultural Research Center, United States Department of Agriculture, College Station, TX USA; 4 Department of Plant Pathology and Microbiology, Texas A&M University, College Station, TX USA

**Keywords:** Computational biology and bioinformatics, Plant sciences

## Abstract

Biological structures interact with light as complex manifolds, yet genomic studies often reduce these phenotypes to scalar traits. Here we resolve the sorghum seed spectral manifold into separable axes corresponding primarily to pigment-associated and structure-associated optical variation. Path analysis indicates that near-infrared (NIR) reflectance is associated primarily with an indirect path through seed mass, supporting partial decoupling from pigmentation-related variation. We identify a chromosome 6 region associated with NIR reflectance and spectral entropy, consistent with a contribution to the physical-structural axis of the seed optical phenotype. Furthermore, in silico perturbation analyses suggest that major axes of seed optics are structured and can be predictably shifted within the observed phenotypic manifold. By linking spectral variation to morphology and genetic association, this work provides a framework for interpreting seed optical phenotypes beyond simple color categories and for studying how light-matter interactions are shaped by plant structure and chemistry.

## Introduction

Seeds are the primary vessels of plant life, engineered by evolution to persist, disperse, and regenerate across heterogeneous environments. Their outer tissues, the seed coat and pericarp, function simultaneously as armor, sensor, and interface: they shield the embryo from pathogens and mechanical stress^1^, regulate gas exchange^2^, and determine how the seed interacts with solar radiation. In staple cereals like sorghum (*Sorghum bicolor* (L.) Moench), maize, and wheat, these interfaces are also focal points of domestication, influencing grain quality, storability, and resistance^3^. Because seed covering structures influence barrier function, mechanical protection, and post-harvest performance, their optical signatures may also capture agriculturally relevant variation that is not apparent from visible color alone. Yet despite their ecological and agricultural centrality, seed optical properties, including how seeds absorb, scatter, and transmit photons across the spectrum, are frequently reduced in genetic studies to one-dimensional descriptors such as “red vs. white” or “light vs. dark.”

In reality, light-matter interactions in biological tissues are inherently high-dimensional. Chemical pigments such as tannins and anthocyanins largely determine absorption in the visible range^4^, whereas physical features—including cuticular waxes, cell wall architecture, and air spaces—contribute to scattering in the near-infrared (NIR)^5,6^. Together, these chemical and physical factors generate a complex spectral signature that reflects aspects of seed chemistry, structure, and physiological state. Hyperspectral imaging has emerged as a powerful tool to capture this complexity, yet its application in plant science has largely remained descriptive or focused on engineering prediction, rather than interrogating the fundamental genetic geometry of the optical space itself.

A critical gap remains in our understanding of how genetic variation shapes this high-dimensional optical phenotype. Genome-wide association studies (GWAS) in sorghum have successfully mapped loci for grain color (*Y* gene, *Tannin1*)^7,8^ and specific phenolic metabolites^9,10^, but they typically treat optical traits as isolated scalar values, ignoring the underlying biophysical constraints. Furthermore, while advances in phenomics have quantified seed morphology at scale^11,12^, these structural traits are rarely integrated with spectral data in a causal framework. Consequently, we still lack a mechanistic framework for understanding how genetic variation contributes to the interplay between pigment biochemistry and structural features in shaping the overall optical phenotype.

Here, we address this challenge by defining the “seed spectral manifold”: a low-dimensional geometric structure that emerges from high-dimensional hyperspectral data. Using a diverse sorghum association panel, we integrate hyperspectral imaging, geometric morphometrics, and dense genotyping to dissect the genetic architecture of this manifold. We ask three fundamental questions: First, is the optical diversity of sorghum seeds structured along coherent biological axes? Second, how do these axes relate to the physical morphology of the seed? Third, can we identify genetic regions associated with pigment-linked and structure-linked optical variation, and can these associations help explain how the spectral manifold may shift under genetic perturbation?

More broadly, this study integrates hyperspectral phenotyping, structural equation modeling (SEM), and genomic association analysis to examine the seed optical phenotype as a structured multivariate trait. By resolving the spectral manifold into pigment-associated and structure-associated axes, we aim to distinguish chemical and physical contributions to seed optics. We further test whether this optical space shows interpretable responses to genetic perturbation in silico, providing a framework for studying seed light-matter interactions beyond conventional color categories.

## Results

### The geometry of the seed spectral phenome

**Fig. 1:**
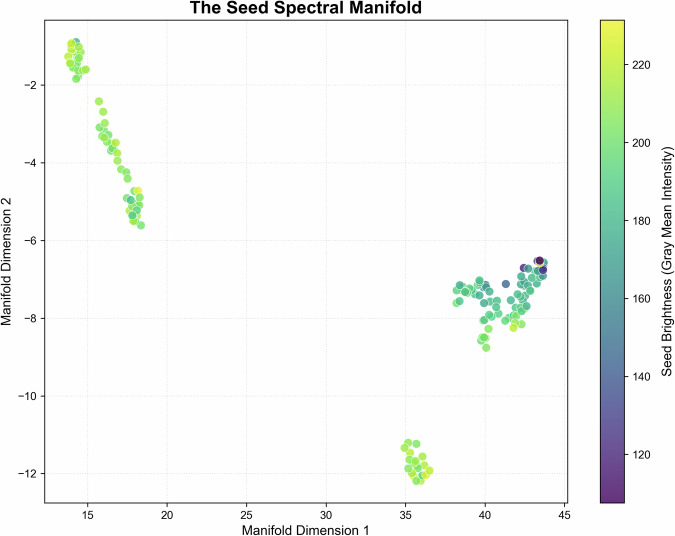
The seed spectral manifold of *Sorghum bicolor.* Two-dimensional embedding of accession-level reflectance spectra across the sorghum panel. Each point represents one accession, and coordinates were derived by UMAP from z-scored reflectance intensities. The manifold forms a continuous trajectory rather than discrete clusters, with point color indicating seed brightness from darker, more absorbing to brighter, more reflective phenotypes.

The optical properties of sorghum seeds are not randomly distributed but occupy a structured, low-dimensional manifold. By embedding high-dimensional reflectance spectra into a latent space using Uniform Manifold Approximation and Projection (UMAP)^13^, we identified a continuous “spectral manifold” that captures the global variation in light-matter interactions (Fig. 1). This manifold was characterized by a primary gradient corresponding to seed brightness, extending from dark, high-absorption phenotypes to bright, high-reflectance phenotypes. Rather than forming discrete classes, the accessions occupied a continuous trajectory, consistent with quantitative variation across the panel.

**Fig. 2:**
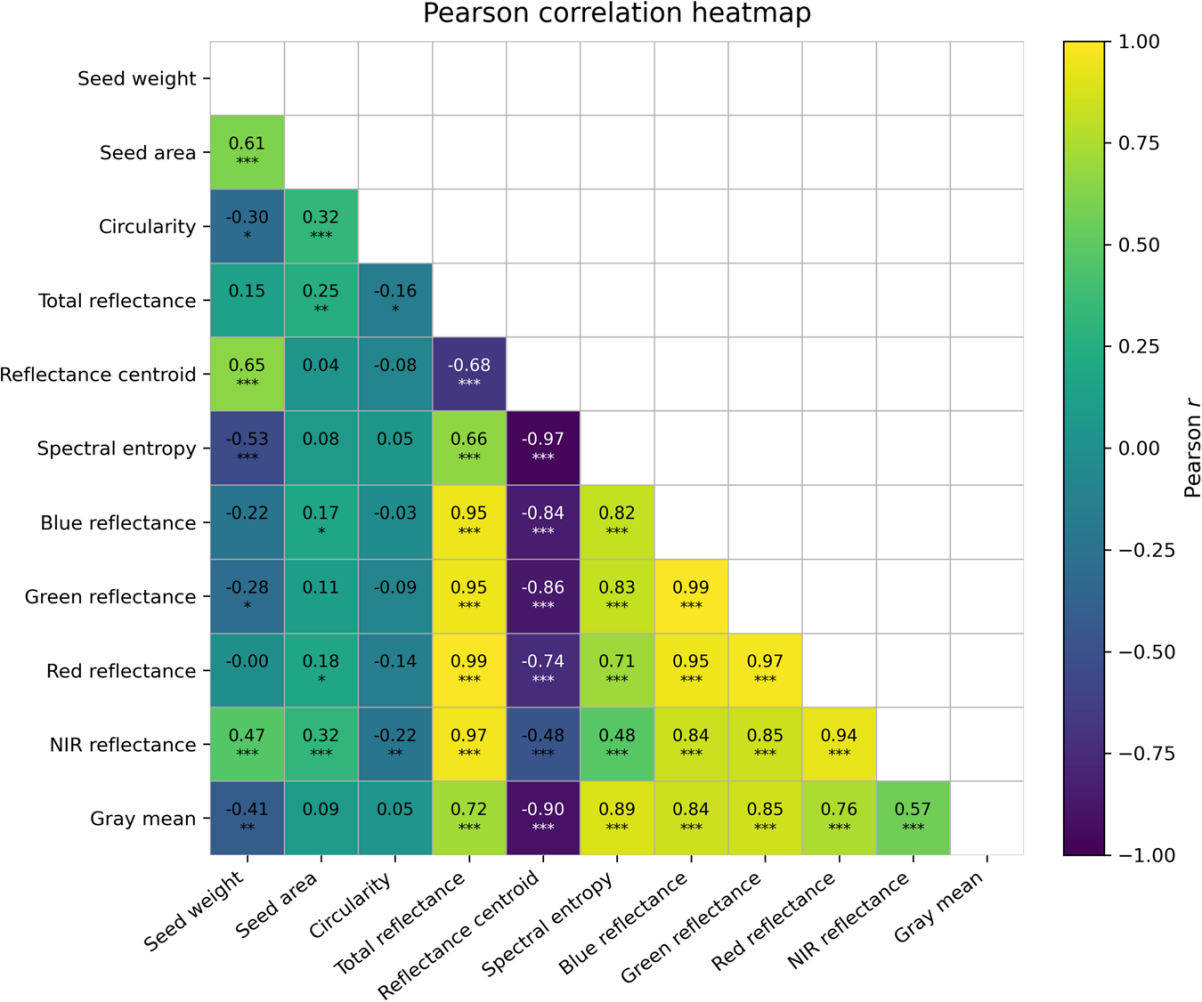
Pairwise correlations between seed morphology and spectral summary traits. Lower triangle shows Pearson correlation coefficients among morphology and spectral traits. Corresponding two-sided *P* values are provided in the source data / supplementary correlation table. Diagonal significance annotations were omitted because self-correlations are not inferentially meaningful. Asterisks indicate two-sided significance levels for Pearson correlations: *P < 0.05, **P < 0.01, and ***P < 0.001.

Reflectance spectra were summarized from segmented seed regions of interest and then aggregated at the accession level; thus, positional heterogeneity within individual seeds contributed to the accession mean profile rather than being analyzed as a separate within-seed factor. Within this manifold, several spectral summary traits were associated with seed morphology. Near-infrared reflectance (${R}_{{NIR}}$) and total reflectance (${R}_{{total}}$) were positively correlated with seed weight and projected area, whereas spectral centroid and peak wavelength were inversely related to brightness-related traits (Fig. 2). Stratifying accessions by brightness further showed that dark seeds had stronger visible-range absorption and a more irregular spectral profile, whereas bright seeds exhibited a flatter and more reflective spectrum across both visible and near-infrared wavelengths (Supplementary Fig. 1). Together, these patterns are consistent with partly separable contributions from pigment-associated absorption and structure-associated scattering.

### Genetic constraints on the optical manifold

**Fig. 3:**
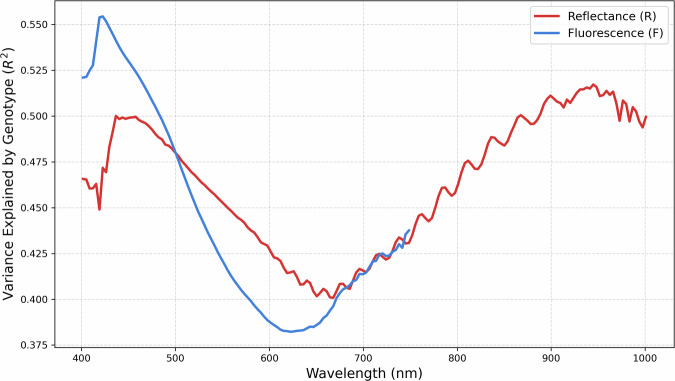
Genomic structure-associated variance across wavelengths. For each wavelength, reflectance intensity was regressed on the first 10 genotype principal components (genotype PC1–genotype PC10), and the coefficient of determination (*R*^2^) is shown as a summary of the proportion of spectral variance aligned with major genomic structure. This metric is not a formal estimate of narrow-sense heritability (*h*^2^) but a descriptive measure of variance explained by major genotype PCs.

To assess how strongly spectral variation tracked major genomic structure, we calculated a genomic structure-associated variance spectrum across wavelengths. The proportion of reflectance variance explained by genotype principal components (PCs) was not uniform across the spectrum but showed distinct peaks in the red and near-infrared regions (Fig. 3). These results indicate that some wavelength regions, particularly those linked to structure-related scattering, were more strongly aligned with major axes of genomic variation than others. Consistent with this pattern, a Mantel test showed a significant positive association between genetic and spectral distances (*r* = 0.42, *P* = 0.001), indicating that divergence in genomic structure was reflected in divergence of the spectral manifold (Supplementary Fig. 2).

### Uncoupling chemical and physical regulation via path analysis

To evaluate whether genotype-associated variation in optics might be mediated through seed structure, we fitted a path model linking genotype PCs to seed area and seed weight, and then to NIR-related traits (Supplementary Table 1). Genotype PC1 and PC2 showed strong standardized associations with both seed area and seed weight (for example, *β* = −0.522 and 0.333 for PC1 and PC2 on seed area, and *β* = −0.467 and 0.658 for PC1 and PC2 on seed weight; all *P* < 10⁻⁶), indicating that these axes capture major structural variation in the panel. In the downstream portion of the model, seed area showed a positive point estimate for NIR reflectance, whereas direct genotype PC-to-NIR paths were comparatively small and not strongly supported statistically. Under the prespecified SEM, these directional patterns are consistent with partial mediation of NIR-related variation through morphology, particularly for genotype PC2, but they should be interpreted cautiously because several downstream coefficients were modest. By contrast, visible color traits showed clearer and more direct correspondence with pigmentation-associated GWAS signals, supporting partial separation between pigment-linked and structure-linked axes of optical variation.

### A chromosome 6 region is associated with the physical-optical interface

**Fig. 4:**
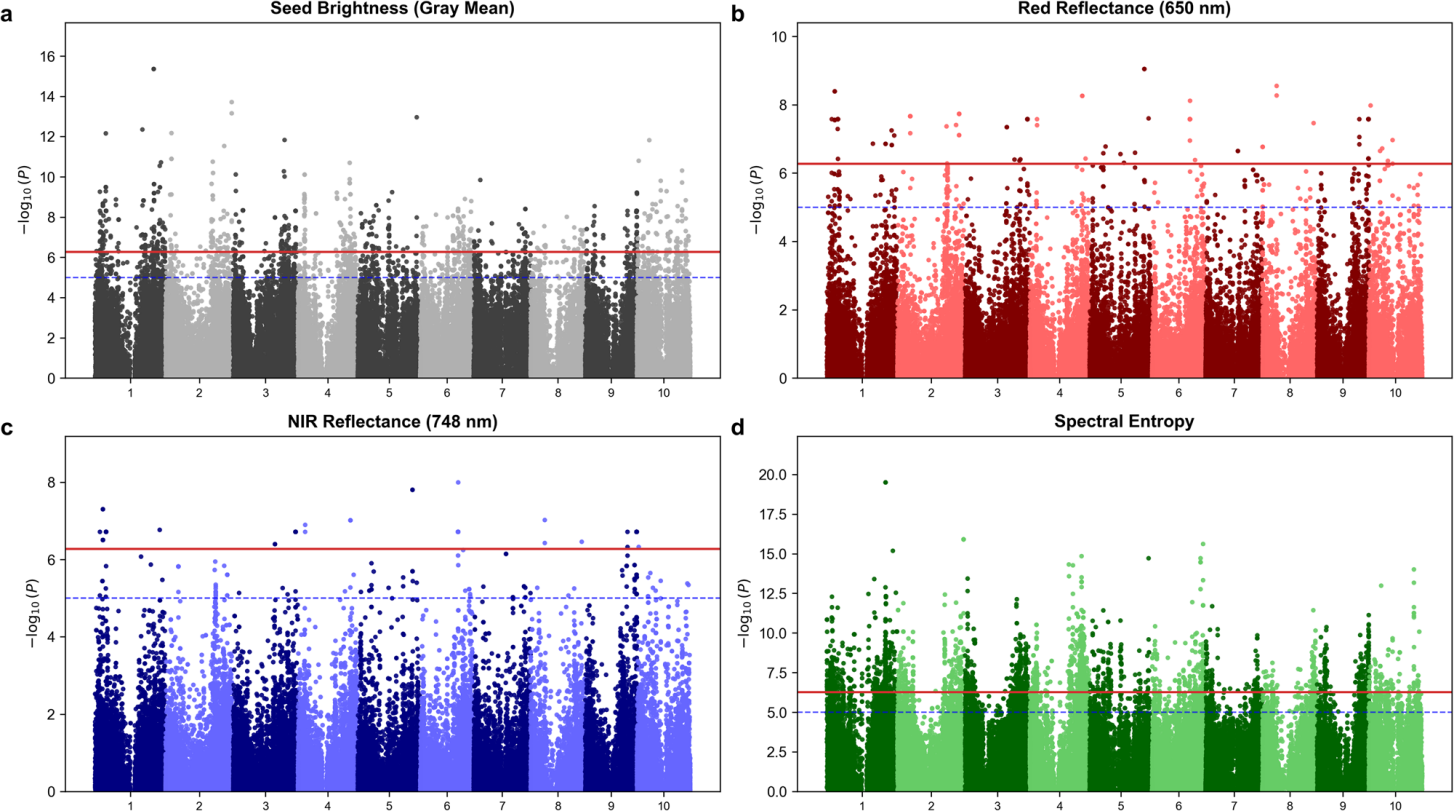
Genome-wide association landscapes for focal optical traits. Manhattan plots for **a** gray mean intensity, **b** reflectance at 650 nm (${R}_{650}$), **c** reflectance at 748 nm (${R}_{748}$), and **d** spectral entropy. The blue dashed line indicates the suggestive threshold ($P < 1\times {10}^{-5}$), and the red solid line indicates the Bonferroni threshold (approximately *P* = $5.4\times {10}^{-7}$). The four traits differ in their apparent association architecture, with broader signal burdens for visible color-related traits and more localized peaks for ${R}_{748}$ and spectral entropy.

Genome-wide association mapping revealed distinct architectures across the focal optical traits. Using GEMMA linear mixed models, we identified multiple associated loci for gray mean intensity, red reflectance at 650 nm (*R*_650_), near-infrared reflectance at 748 nm (*R*_748_), and spectral entropy at a suggestive threshold of $P < 1\times {10}^{-5}$ (Fig. 4; Table 1; Supplementary Data 1). Significant SNPs were grouped into operational QTL intervals using a ± 250 kb window, as described in Methods. Overall, visible color-related traits showed broader and more polygenic association landscapes, whereas *R*_748_ and spectral entropy exhibited more localized peaks.

**Table 1:** Representative associated loci for focal optical traits in sorghum seeds

Trait	Chr	Lead SNP	Position (Mb)	Lead *P*	Representative nearby gene(s)
Gray mean	3	S3_52269871	52.27	2.36 × 10⁻¹²	*Sobic.003G197400*
Gray mean	6	S6_44399399	44.4	1.03 × 10⁻⁸	*Sobic.006G078200*
*R*_650_	1	S1_67303813	67.3	7.12 × 10⁻¹³	*Sobic.001G385500*
*R*_650_	9	S9_6640622	6.64	2.26 × 10⁻¹²	*Sobic.009G062600*

**Fig. 5:**
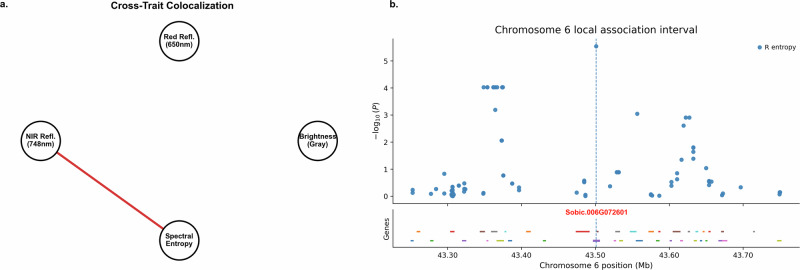
A chromosome 6 interval associated with structure-linked optical traits. **a** Cross-trait colocalization network showing overlap among QTL intervals across focal spectral traits. A chromosome 6 node at ~43.5 Mb links ${R}_{748}$ and spectral entropy. **b** Local association view of the chromosome 6 interval, showing the lead signal, the ±250 kb operational window, and annotated nearby genes within the region.

Lead SNPs are shown for selected representative loci associated with gray mean intensity and reflectance at 650 nm (*R*_650_), based on the summarized peak table used for main-text presentation. Nearby annotated genes are listed for regional interpretation only. Full locus summaries, including the chromosome 6 interval associated with *R*_748_ and spectral entropy, are provided in Supplementary Data 1 and Fig. 5.

Cross-trait colocalization highlighted a chromosome 6 interval at approximately 43.5 Mb (lead SNP: S6_43500928) that was associated with both ${R}_{748}$ and spectral entropy (Fig. 5; Table 1). Revision-stage local association analysis showed that this signal lies within a broader associated interval that does not overlap the canonical pigmentation genes *Y* or *Tannin1* and that contains multiple annotated genes within the ±250 kb operational window. Among nearby candidates, *Sobic.006G072601* encodes a DUF6598-domain protein and remains one plausible gene of interest, but the present data do not isolate a single causal gene. Because ${R}_{748}$ and spectral entropy both capture aspects of structure-linked optical variation, this interval is consistent with a candidate contribution to the physical-structural axis of the seed optical phenotype. Additional local linkage disequilibrium (LD) and haplotype analyses are provided in Supplementary Fig. 3 and Supplementary Fig. 4.

### In silico design of the spectral phenome

**Fig. 6:**
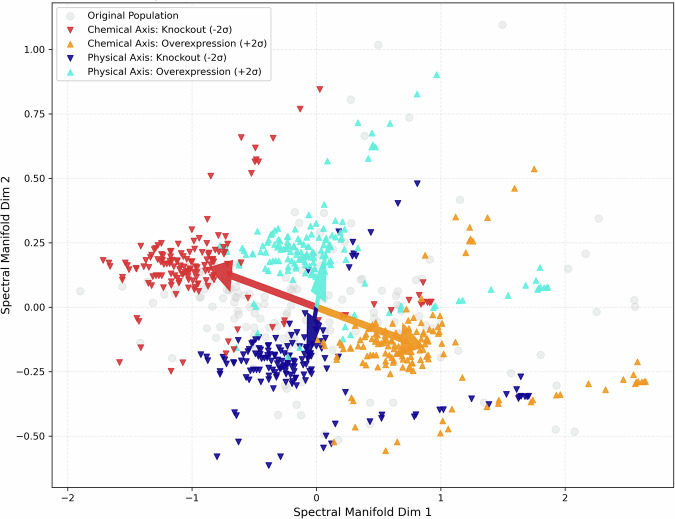
In silico evolution of the seed spectral manifold. Projected deformation of the spectral manifold under virtual genetic perturbation. **a** Original manifold (gray). **b** Shift under in silico knockout (red) and overexpression (blue) of genotype PC1 (chemical-associated axis) and PC2 (structure-associated axis). Arrows indicate contrasting trajectories of phenotypic change, consistent with partial separation between pigment-associated and structure-associated components of the seed optical phenome.

Finally, we asked whether the spectral manifold would respond in structured ways to perturbation of genotype-associated axes in a multivariate model. By altering specific genotype PCs, we simulated hypothetical shifts in manifold position under directional genetic change. Perturbation of genotype PC1 compressed the manifold primarily along the brightness-related axis, whereas perturbation of genotype PC2 produced a distinct oblique shift affecting NIR-related variation and spectral shape (Fig. 6). These contrasting trajectories are consistent with partial separation between pigment-associated and structure-associated components of the seed optical phenome, although the simulations should be interpreted as model-based projections rather than direct predictions of breeding outcomes.

### Genetic control of seed optical uniformity

Beyond average optical properties, we investigated the genetic basis of inter-seed optical uniformity. The GWAS for NIR reflectance uniformity (${R}_{750{Std}}$) identified a highly significant peak on chromosome 4 (Supplementary Fig. 5a). The lead SNP colocalized with *Sobic.004G120000* (expansin-A24) and *Sobic.004G269400* (pectin acetylesterase 8). An independent peak on chromosome 1 ( ~ 11.1 Mb) mapped to a region containing a CSC1-like $C{a}^{2+}$ channel (*Sobic.001G140000*) and a bHLH transcription factor (*Sobic.001G140800*).

For metabolic heterogeneity, defined by the skewness of blue fluorescence ($F{450}_{{Skew}}$), we detected a strong signal on chromosome 8 (Supplementary Fig. 5b). The lead SNP resides near *Sobic.008G147101* (MYB-like transcription factor). Additional peaks on chromosomes 7 ( ~ 2.8 Mb) and 9 ( ~ 9.0 Mb) co-localized with *Sobic.007G032100* (HD-ZIP transcription factor) and *Sobic.009G073100* (ZIP zinc transporter), respectively. Loci on chromosomes 1 (~ 68 Mb) and 3 (~ 61.8 Mb) further implicated galactinol synthase and galacturonosyltransferase candidates.

## Discussion

In this study, we treat seed optics not as a single “color” trait, but as a high-dimensional phenotype emerging from the interaction between genes, structure, and light. We conceptualize this phenotype as a spectral manifold, a constrained phenotypic space shaped by the biological limits of gene–structure interactions. Just as a seed cannot assume an arbitrary shape, its optical properties are restricted to specific trajectories defined by its physical architecture. By integrating hyperspectral imaging, morphology, population genomics, and mixed-model GWAS, we show that the sorghum seed spectral manifold is (i) low-dimensional but structured by major genomic variation, (ii) organized along separable axes corresponding broadly to pigment chemistry and physical structure, and (iii) responsive to in silico genetic perturbation. These results support the interpretation of seed color and reflectance as multivariate phenotypes shaped by both chemistry and structure, rather than as simple visible descriptors alone.

The collapse of hyperspectral data onto a smooth, low‑dimensional manifold tracks genome-wide variation, reflecting the tight genetic coordination of seed coat development^1,14^. This continuous trajectory, from dark, absorbing to bright, scattering phenotypes, may reflect the quantitative accumulation of structural components and chemical deposits known to regulate dormancy and longevity^11,15^.

A key insight is the decomposition of optical variation into “chemical” and “physical” components. The chemical axis is driven by pigmentation. Consistent with studies in soybean and other legumes, darker seeds accumulate higher concentrations of phenolic compounds and tannins, which drastically alter water permeability and susceptibility to imbibitional injury^2,16^. Our GWAS supports the interpretation that brightness and visible-range traits are linked to pigmentation-related loci, consistent with the known contribution of flavonoid pathway regulators such as *TAN1* and *TAN2* to seed coat chemistry^7,8^.

Consistent with Mie scattering theory^5,6^, we found that NIR reflectance is shaped by tissue bulk properties and structural architecture. While previous studies often treated structural variation as a source of distortion in NIRS measurements^3,17^, our results suggest that such variation can itself be interpreted as a biologically meaningful phenotype. The association between NIR scattering and seed mass indicates that this optical axis captures structure-associated variation in the seed, consistent with prior morphological findings^12^. Furthermore, correlations between spectral entropy and circularity suggest that surface curvature and other unmeasured architectural features may act as secondary modulators of the light path.

Our mixed-model GWAS highlighted a chromosome 6 interval (~ 43.5 Mb) that was associated with both NIR reflectance and spectral entropy, two traits that capture aspects of structure-linked optical variation. Revision-stage local association, LD, and haplotype analyses further supported the regional coherence of this signal, but they did not isolate a single causal gene within the interval. Multiple annotated genes fall within the operational window, and one nearby candidate, *Sobic.006G072601*, encodes a DUF6598-domain protein. Although this gene remains functionally uncharacterized in sorghum, the interval as a whole is consistent with a contribution to seed structural optics.

One plausible interpretation is that genetic variation within this region influences seed coat architecture or cell wall organization in ways that alter near-infrared scattering. This interpretation is compatible with prior work showing that cell wall-associated proteins, including thaumatin-like proteins and related defense-associated factors, can influence permeability, rigidity, and surface properties of plant tissues^18–22^. In optical terms, such changes could modify refractive index contrast and photon scattering at tissue interfaces^5^. However, our data do not establish that *Sobic.006G072601* is the causal gene, nor do they demonstrate a specific TLP-like mechanism. We therefore interpret the chromosome 6 signal as a candidate structural-optics interval whose biological basis remains to be resolved by future functional and histological work.

The in silico perturbation analysis suggests that the spectral manifold can respond in structured ways to changes in genotype-associated axes. This extends conventional GWAS by framing optical phenotypes within a geometric view of phenotypic space. In practical terms, the results imply that some components of seed optical variation may be shifted independently of others, raising the possibility that structure-associated and pigment-associated traits could be at least partially decoupled. Because the seed coat functions as a primary physical interface between the embryo and the environment^1^, this framework may prove useful for future efforts to connect hyperspectral phenotypes with seed covering properties, although such applications will require further validation.

Our analysis of inter-seed variation provides a window into the developmental stability, or canalization, of seed optical traits. The association of NIR uniformity (${R}_{750{Std}}$) with a region containing an expansin-A24 candidate is consistent with the possibility that cell wall expansion and tissue organization contribute to between-seed variation in scattering^23^. Because expansins regulate cell wall loosening during growth, allelic differences in this region could plausibly influence the uniformity of seed tissue architecture during filling. Such variation may in turn contribute to differences in density or air-space distribution among seeds, which would be expected to affect NIR scattering. The additional ${R}_{750{Std}}$ locus on chromosome 1, which contains a CSC1-like Ca2+ channel and a bHLH transcription factor, further suggests that mechanosensitive signaling and transcriptional regulation may contribute to this structural variability^24^.

Similarly, the association of fluorescence skewness (${F}_{450{Skew}}$) with a MYB transcription factor candidate is consistent with the idea that inter-seed variation in fluorophore accumulation has a genetic component. Because MYB factors commonly regulate flavonoid and phenylpropanoid pathways^25–27^, variation in this region could plausibly influence the asymmetry of fluorescence distributions across seeds within an accession. Additional ${F}_{450{Skew}}$ loci extend this interpretation: an HD-ZIP transcription factor on chromosome 7 links the signal to epidermal and cuticle differentiation, whereas a ZIP-family zinc transporter on chromosome 9 points to a possible role for micronutrient homeostasis. Together with candidates involved in raffinose and pectin metabolism, these loci suggest that both structural and metabolic variability in seed optics may be shaped by genes involved in cell wall biology, signaling, and stress-related metabolism.

Our study is limited by the absence of direct histological validation for the chromosome 6 locus. Future research should utilize CRISPR or NILs to confirm whether allelic variation at this locus alters cell wall thickness or composition, as seen with *GhTLP1* in cotton^19^. Additionally, since seed coat permeability and optical properties are sensitive to environmental moisture^28^, multi-environment trials are necessary to partition genotype-by-environment interactions. Nevertheless, this work provides a proof-of-concept that the seed spectral manifold can be interpreted as an integrated readout of genes, structure, and light interaction. More broadly, it suggests that optical measurements may provide a non-destructive window into biologically meaningful variation in seed architecture and composition.

## Methods

### Plant material and experimental design

A diversity panel consisting of 162 *Sorghum bicolor* (L.) Moench accessions originating from Senegal was initially employed in this study. The germplasm collection and maintenance were performed as previously described^12^. Seeds were obtained from verified stocks maintained by the USDA-ARS Plant Genetic Resources Conservation Unit. Seed morphological traits, including projected area (mm^2^), circularity, length, width, and seed weight, were retrieved from our previously published phenotypic dataset^12^, where high-resolution RGB imaging (SmartGrain ver. 1.3)^29^ and gravimetric measurements were conducted. For the current study, these pre-existing morphological data were merged with the newly acquired hyperspectral datasets to construct a unified “seed phenome” matrix. Following data quality control, accessions with incomplete genotypic or hyperspectral data were excluded, resulting in a final set of 156 accessions for all downstream genomic and integrative analyses.

### Hyperspectral imaging system

Hyperspectral images of seed samples were acquired using a custom-built line-scan visible and near-infrared (VNIR) imaging system covering wavelengths from 400–1000 nm. The setup was adapted from a previously developed system^30^. The HSI system consisted of a 12-bit complementary metal-oxide-semiconductor (CMOS) detector array (Hyperspec MV.C VNIR, Headwall, Bolton, MA, USA) with 1024 spatial pixels, block Offner spectrograph, a 5 mm focal length wide-angle objective lens (Edmund Optics, Barrington, NJ, USA), a translational stage, and a display unit. The spectral resolution was approximately 6.0 nm (full width at half maximum). For this study, data were acquired in two distinct hyperspectral modes:

1. Reflectance Mode: Seeds were illuminated by a 150-W DC quartz-tungsten halogen lamp (Dolan Jenner, Boxborough, MA, USA) via fiber optic line guides to ensure uniform broadband illumination across the instantaneous field of view (IFOV).
2. Fluorescence Mode: Excitation was provided by a pair of custom UV-A LED units (365 nm; LedEngin, San Jose, CA, USA) to induce autofluorescence from seed coat phenolic compounds.

### Image acquisition and calibration

Seed samples were placed in the sample holder and transferred to the conveyor unit for HSI image acquisition. To improve signal quality, exposure times were set to 150 ms for reflectance imaging and 250 ms for fluorescence imaging, with a scanning step size of 0.2 mm. The programmable precision positioning table (Xslide, Velmex Inc., Bloomfield, NY, USA) moves the samples towards the FOV of the camera. Scanning was performed line by line in both reflectance and fluorescence modes and the data was stored in a 3D format for further analysis (Supplementary Fig. 6). To calibrate the raw reflectance images, white and dark HSI reference images were collected immediately after scanning. For fluorescence images, only the dark reference was used. The dark image was acquired by covering the camera lens, while the white reference image was obtained using a Teflon board with reflectance greater than 99%. Calibration of the raw hyperspectral images was performed using Equation 1. A similar calibration equation was applied to fluorescence images, excluding the white reference subtraction.

$${I}_{R\left({calibrated}\right)}=\frac{{I}_{R}-{I}_{D}}{{I}_{W}-{I}_{D}}\times 100$$

where ${I}_{R\left({calibrated}\right)}$ is the corrected image, ${I}_{R}$ is the raw sample image, ${I}_{D}$ is the dark reference image, and ${I}_{W}$ is the white reference image.

Mean spectra for each accession were extracted by spatially segmenting seed regions from the background using an image thresholding method^30^. This involved generating a binary mask in which seed pixels were assigned a value of 1 and background pixels a value of 0. The binary mask was then applied to the calibrated images to remove the background pixels, leaving only the seed region. A region-of-interest (ROI) approach was subsequently used to extract the spectral data for each seed, which was essential for downstream modeling. The extracted spectral data were then trimmed to effective wavelength ranges to ensure high signal quality: reflectance spectra were retained across 400–1000 nm, while the fluorescence spectra were restricted to 400–750 nm to minimize noise and excitation artifacts.

### Spectral feature extraction and manifold learning

To quantify the high-dimensional optical properties, we derived physics-based summary statistics from the calibrated spectra. Total reflectance (${R}_{{total}}$) and total fluorescence (${F}_{{total}}$) were calculated as the integral of the spectral curve. Spectral entropy (*S*), a measure of waveform complexity, was computed using Shannon’s formula on the normalized spectral distribution^31^. Additionally, the spectral centroid (${\lambda }_{c}$) was calculated as the intensity-weighted mean wavelength. To visualize the global geometry of the seed optical phenome, we applied Uniform Manifold Approximation and Projection (UMAP)^13^ to the standardized reflectance matrix, generating two latent coordinates (${R}_{{UMAP}1}\& \,{R}_{{UMAP}2}$) that summarized the low-dimensional geometry of the spectral manifold. The embedding was computed using n_neighbors = 15, min_dist = 0.1, and the Euclidean distance metric. Qualitative features of the manifold, including the continuous transition from darker to brighter seeds, were robust across a range of n_neighbors values (10–50) and random seeds (data not shown).

### Pairwise correlation analysis

Pairwise relationships among morphology and spectral summary traits were quantified using Pearson correlation coefficients (*r*). Two-sided significance tests were computed for each pairwise comparison, and the resulting *P* values were used only for off-diagonal entries. Because self-correlations are not inferentially meaningful, diagonal significance annotations were omitted from the correlation heatmap. For figure clarity, only the lower triangle of the correlation matrix was displayed in the revised heatmap.

### Genotyping and population structure

Genotyping-by-sequencing (GBS) data were obtained from an integrated sorghum SNP dataset based on the *Sorghum bicolor* reference genome v3.1.1 (Phytozome v13)^32^. The original dataset was generated and processed as described in our previous association studies^33^. For downstream analyses, genotype calls were converted to PLINK-compatible formats when needed. For the primary association mapping of seed-average spectral traits, we applied a quality-control filter of minor allele frequency (MAF) > 0.05 and missing rate < 10%, yielding a filtered set of 93,174 SNPs for common-variant association testing^34–37^. For the targeted analysis of inter-seed optical heterogeneity, we used a broader marker set of 195,661 SNPs without the MAF filter in order to retain variants that might contribute to within-accession distributional traits.

To summarize population structure, SNP dosages were mean-centered by marker and principal components (PCs) were computed by singular value decomposition (SVD) of the genotype matrix. The first 10 genotype PCs were retained because they captured the dominant structure of the panel and together explained most of the sample-space variance; the corresponding scree plot is provided in Supplementary Fig. 7. These PCs were used as covariates in population-structure correction and as genotype-level predictors in integrative path analyses.

### Path analysis and structural equation modeling

To evaluate a hypothesized directional relationship from genotype to morphology to optics, we constructed a structural equation model (SEM)^38^. We hypothesized that genotypic variation (genotype PCs) primarily constrains morphological traits (seed weight, projected area), which in turn impose biophysical constraints on optical properties (NIR reflectance, spectral entropy).

Path coefficients were estimated using two-stage least squares regression, allowing us to partition genotype-associated effects into direct components aligned with pigment-related traits and indirect components mediated through morphology-associated structural features.

### Genome-wide association studies

Genome-wide association analyses were performed using univariate linear mixed models (LMMs) implemented in GEMMA^39^ in order to control for relatedness and residual population structure. A centered kinship matrix was estimated from the filtered SNP panel using the standard centered relatedness algorithm (-gk 1), and association testing was conducted using Wald statistics (-lmm 4). For the primary seed-average spectral analyses, we evaluated four focal traits: gray mean intensity, red reflectance at 650 nm (${R}_{650}$), near-infrared reflectance at 748 nm (${R}_{748}$), and spectral entropy. Associated SNPs were summarized using a suggestive significance threshold of $P < {10}^{-5}$.

To summarize neighboring association signals at the locus level, significant SNPs were grouped into operational QTL intervals using a ± 250 kb window. This interval was used to consolidate nearby peaks for interpretation rather than to define causal boundaries. To contextualize this window size, we calculated empirical linkage disequilibrium (LD) decay in the filtered SNP panel; LD declined to approximately *r*^2^ = 0.2 at ~112.5 kb (Supplementary Fig. 8), indicating that the ±250 kb grouping window was conservative for regional summarization. Cross-trait colocalization was defined where QTL intervals from different traits overlapped. No additional LD pruning was applied to the primary GEMMA scans beyond the stated SNP quality filters; instead, LD pruning summaries were generated post hoc during revision to characterize marker redundancy and local genomic structure.

For revision-stage interpretation of the chromosome 6 signal, we additionally computed local LD summaries and haplotype-level phenotype contrasts within the associated interval. Candidate genes were annotated by intersecting QTL intervals with the *Sorghum bicolor* reference annotation (Phytozome v13)^32^. These annotations were used to identify genes within associated intervals, but nearest-gene proximity was not interpreted as proof of causality.

### In silico spectral evolution

To test the designability of the seed phenome, we moved from static association to dynamic prediction. We constructed a multivariate model acting as a ‘transfer function’ between the genomic landscape (genotype PCs) and the optical manifold (spectral PCs). We then simulated virtual evolutionary trajectories by mathematically perturbing specific genetic axes by $\pm 2\sigma$, mimicking the effects of strong directional selection (‘knock-out’ vs. ‘over-expression’ scenarios). When projected back onto the manifold, these in silico perturbations revealed coherent, axis-specific deformations: manipulating the chemical axis compressed the manifold along the brightness gradient, whereas modulating the physical axis induced a distinct trajectory affecting NIR scattering. This suggests that the spectral manifold is not fixed and may be shifted along interpretable genetic axes in the model.

### Quantification of inter-seed optical heterogeneity

To investigate the genetic basis of optical uniformity within accessions, we derived inter-seed distributional metrics from hyperspectral measurements of individual seeds rather than using accession-level mean spectra alone. For each accession, we quantified the standard deviation (Std) and skewness of selected wavelength-specific signals across seeds. We focused in particular on the Std of near-infrared reflectance at 750 nm (${R}_{750{Std}}$) as an indicator of variability in structure-related scattering, and the skewness of blue fluorescence at 450 nm (${F}_{450{Skew}}$) as a descriptor of asymmetry in accession-level fluorescence distributions. These inter-seed traits were analyzed using the same GEMMA-based mixed-model framework^39^ described above, except that the broader 195,661-SNP set was retained in order to preserve markers potentially relevant to within-accession heterogeneity.

## Supplementary information

Supplementary Information

Supplementary Data

## Data Availability

The seed morphology phenotype data analyzed in this study were previously published and are openly available in Supplementary Data 1 of Ahn et al. (Plants 12, 2344; 2023) via 10.3390/plants12122344. The genotypic variant (SNP) data generated and analyzed during the current study have been deposited in the European Variation Archive (EVA) at EMBL-EBI under project accession number PRJEB108543 (https://www.ebi.ac.uk/eva/?eva-study=PRJEB108543). Additionally, these SNP datasets are accessible through the Figshare repository via 10.6084/m9.figshare.30877697. Curated spectral trait matrices, inter-seed optical heterogeneity traits, GWAS summary tables, and candidate-gene summaries supporting the findings of this manuscript are provided in Supplementary Data 1 associated with this article. The custom code used for spectral decomposition and statistical validation is openly available at: https://github.com/EJSAHN/sorghum-seed-spectral-manifold. All other data and materials supporting this study are available from the corresponding author upon reasonable request.
