# Supplementary material for: Decoupling the chemical and physical origins of the seed spectral manifold in sorghum
Source: NPJ Syst Biol Appl. 2026 May 11;12:110. doi: 10.1038/s41540-026-00728-w (PMC13381802; doi:10.1038/s41540-026-00728-w)
Supplement: Supplementary file 1 — Supplementary Information [file 41540_2026_728_MOESM1_ESM.pdf]

**Response trait**	**Predictor**	**Standardized *β***	**SE**	***t***	***P* value**
Seed area	Genotype PC1	−0.522	0.059	−8.91	1.34×10^{-15}
Seed area	Genotype PC2	0.333	0.059	5.67	6.76×10^{-8}
Seed area	Genotype PC3	0.289	0.059	4.93	2.11×10^{-6}
Seed weight	Genotype PC1	−0.467	0.081	−5.79	3.74×10^{-7}
Seed weight	Genotype PC2	0.658	0.081	8.09	7.09×10^{-11}
Seed weight	Genotype PC3	0.193	0.082	2.36	2.21×10^{-2}
NIR reflectance	Seed area	0.25	0.149	1.68	1.00×10^{-1}
NIR reflectance	Seed weight	0.143	0.217	0.66	5.11×10^{-1}
NIR reflectance	Genotype PC1	−0.232	0.153	−1.51	1.37×10^{-1}
NIR reflectance	Genotype PC2	0.091	0.176	0.52	6.07×10^{-1}
NIR reflectance	Genotype PC3	0.112	0.126	0.89	3.78×10^{-1}
Total reflectance	Seed area	0.281	0.165	1.71	9.37×10^{-2}
Total reflectance	Seed weight	−0.150	0.24	−0.63	5.33×10^{-1}
Total reflectance	Genotype PC1	−0.242	0.17	−1.43	1.61×10^{-1}
Total reflectance	Genotype PC2	−0.015	0.195	−0.08	9.38×10^{-1}
Total reflectance	Genotype PC3	0.164	0.14	1.17	2.47×10^{-1}

Supplementary Table 1. Path analysis linking genotype principal components, seed morphology, and NIR-related optical traits. Standardized coefficients are shown for the prespecified SEM relating genotype PCs to seed area and seed weight, and subsequently to NIR and total reflectance. The results support strong genotype–morphology associations and directional evidence consistent with partial morphology-mediated contributions to NIR variation, although several downstream coefficients were modest and should be interpreted cautiously.

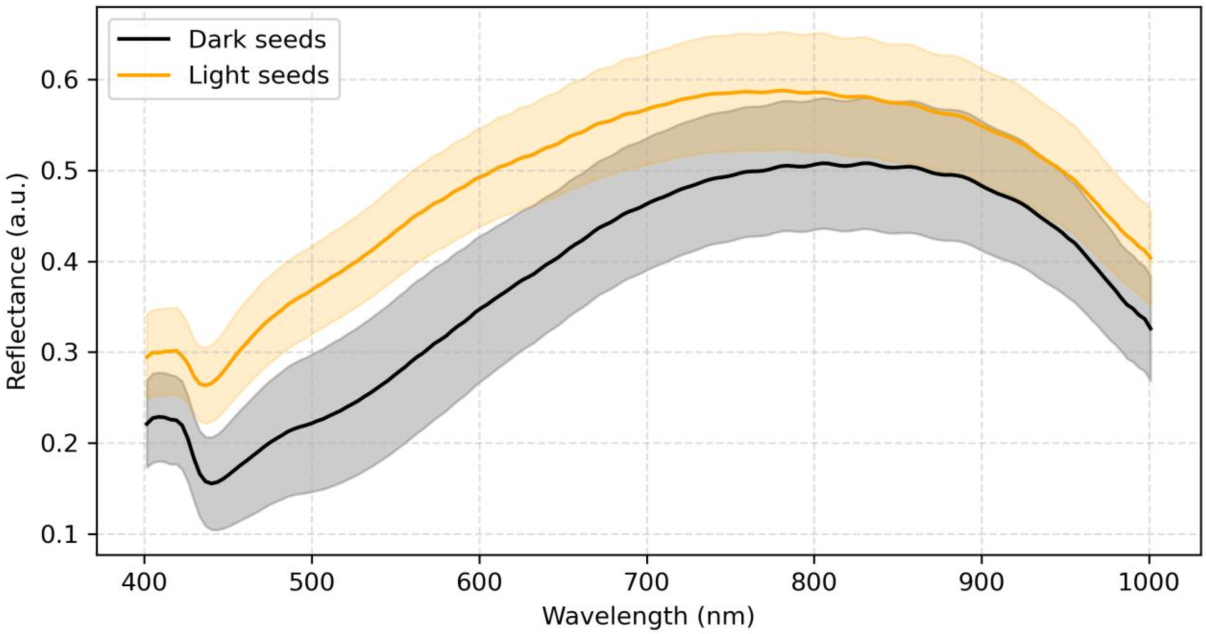

Supplementary Figure 1. Divergent spectral profiles of dark and bright seeds. Comparison of mean reflectance spectra between the darkest (Q1) and brightest (Q4) quartiles of accessions. Dark seeds exhibit strong absorption in the visible range and complex waveform entropy, while bright seeds display a flattened, high-reflectance profile across the visible and NIR regions. Shaded areas represent ±1 S.E.

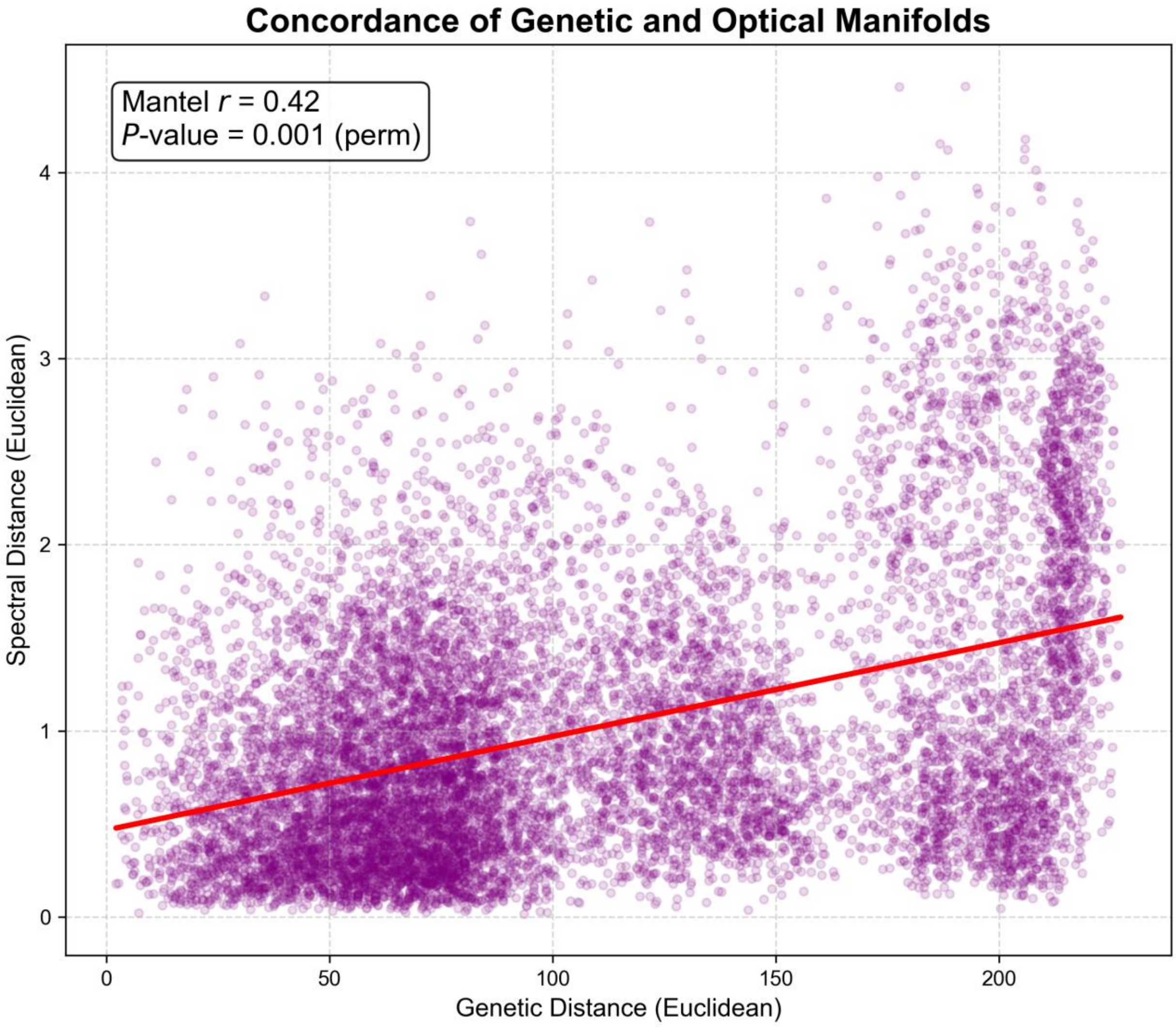

Supplementary Figure 2. Concordance of genetic and optical manifolds. Scatterplot showing the relationship between pairwise genetic distance (Euclidean distance in genotype PC space) and spectral distance (Euclidean distance in Spectral PC space). A significant positive correlation (Mantel $r = 0.42$, $P = 0.001$) indicates that spectral divergence tracks major genomic divergence across the panel.

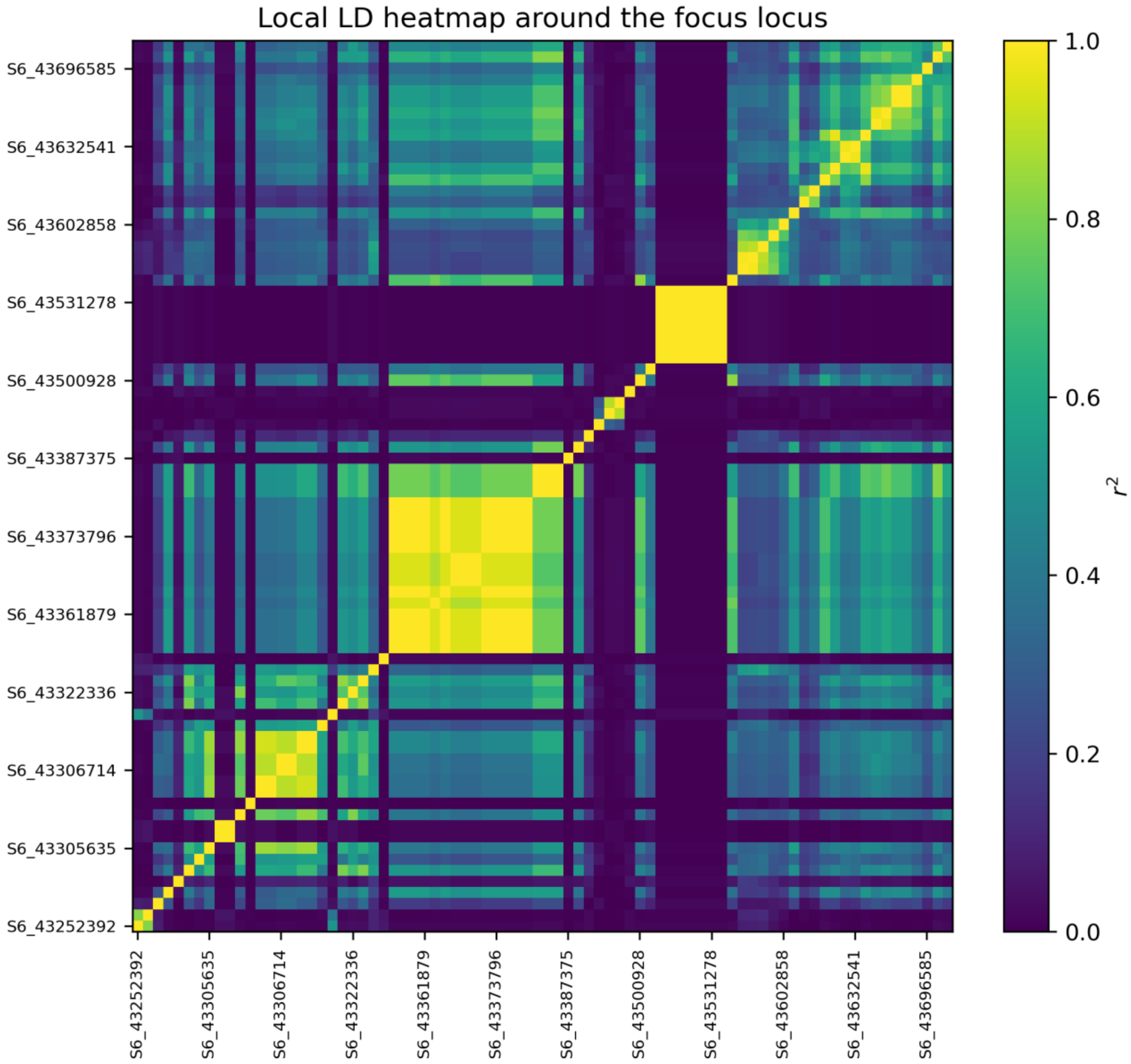

Supplementary Figure 3. Local linkage disequilibrium structure across the chromosome 6 association interval. Pairwise LD among markers surrounding the chromosome 6 signal associated with R_{748} and spectral entropy is shown as a local heatmap. This analysis provides regional support for the associated interval but does not isolate a single causal gene within the window.

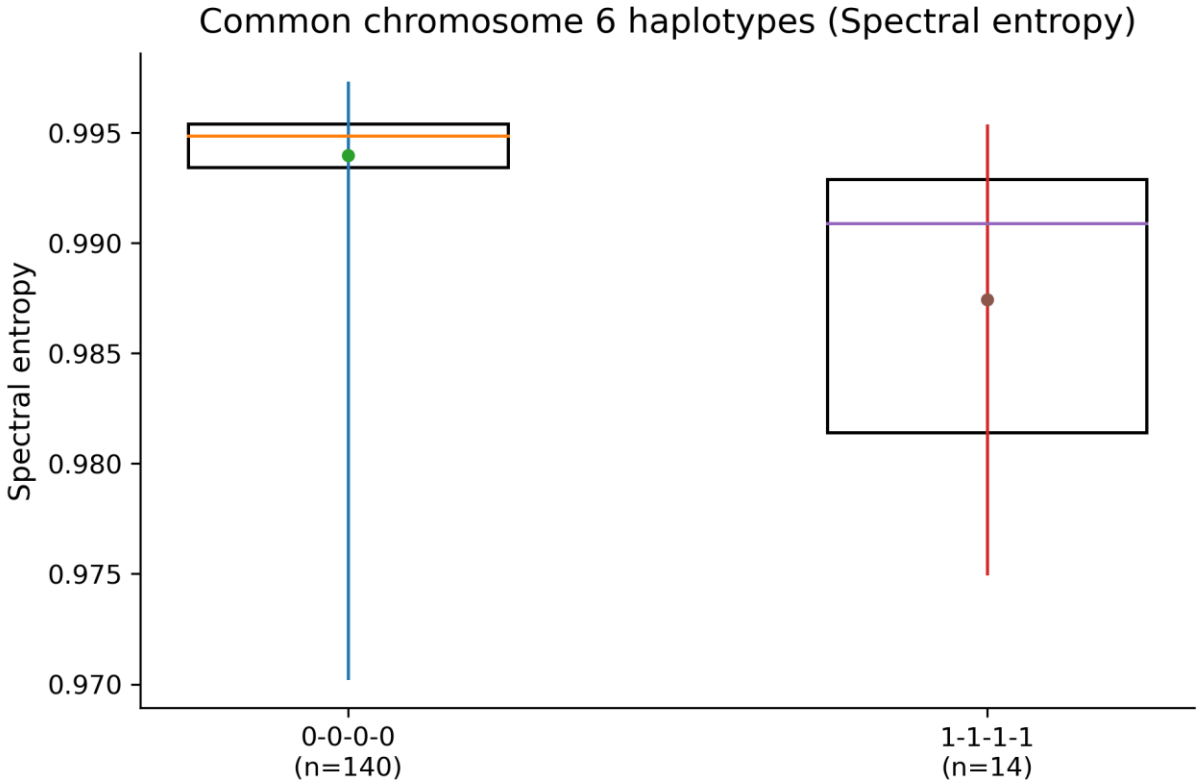

Supplementary Figure 4. Haplotype-level distribution of a structure-linked optical trait across the chromosome 6 interval. Accessions were grouped by local multi-SNP haplotypes defined from markers in the chromosome 6 associated interval, and the distribution of the focal optical phenotype is shown for each haplotype class. This analysis is intended as supportive regional evidence rather than definitive fine-mapping of a causal variant. The haplotype labels indicate binary allele patterns across the local marker set, where 0 and 1 denote the two observed allelic states at each marker.

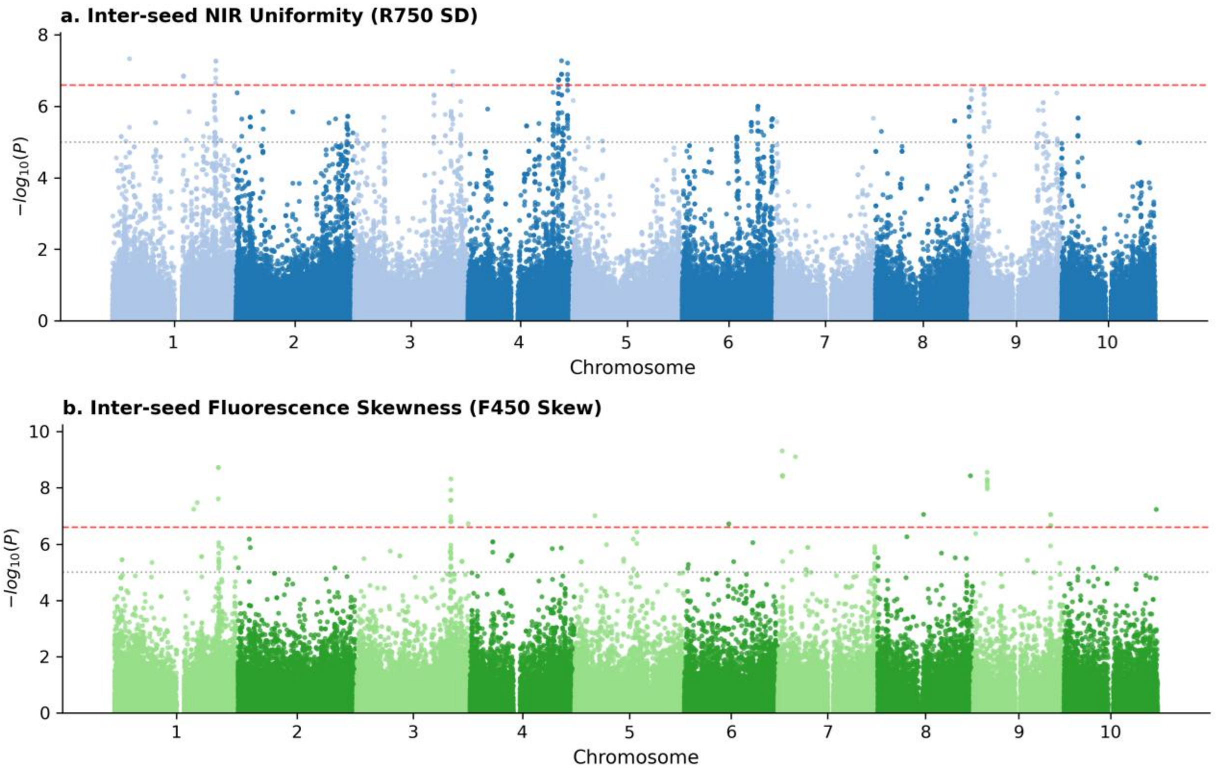

Supplementary Figure 5. Genome-wide association for seed optical uniformity. Manhattan plots showing genetic loci associated with inter-seed variability within accessions. (a) Inter-seed standard deviation of NIR reflectance ($R_{750\ Std}$), representing the uniformity of structural light scattering. Peaks indicate loci that control the developmental consistency of the seed coat structure. (b) Skewness of blue fluorescence ($F_{450\ Skew}$), reflecting the heterogeneity of seed metabolic or defense states. The dashed red line indicates the Bonferroni-corrected significance threshold, and the dotted gray line indicates a suggestive threshold ($P < 1 \times 10^{-5}$). Major peaks include an expansin-A24 locus on Chromosome 4 and a CSC1-like/bHLH locus on chromosome 1 for $R_{750\ Std}$, as well as MYB, HD-ZIP, and ZIP-type transporter loci for $F_{450\ Skew}$

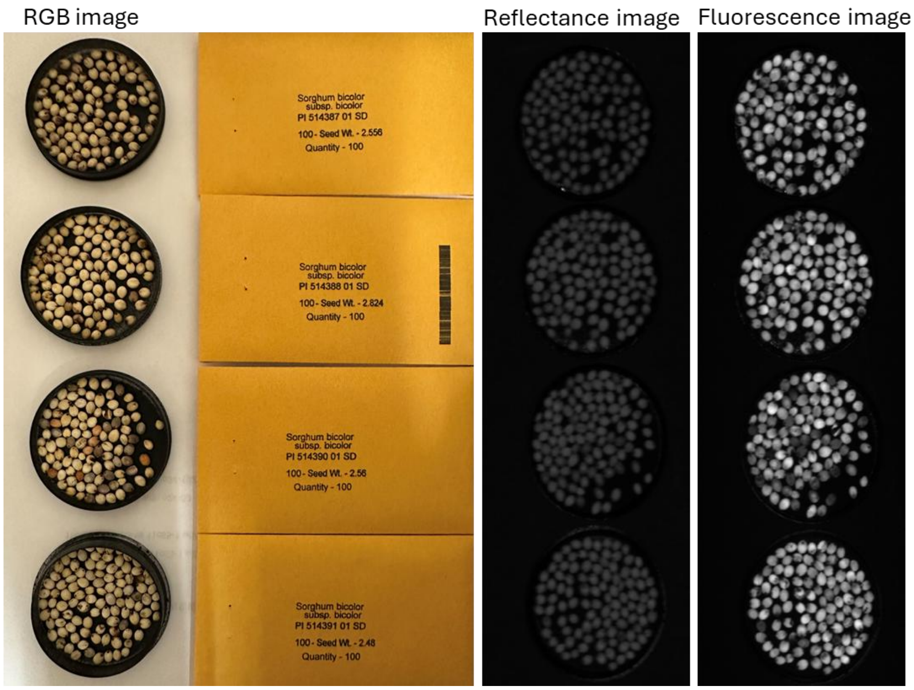

Supplementary Figure 6. Hyperspectral imaging acquisition setup showing seed sample arrangement and representative images in reflectance and fluorescence modes.

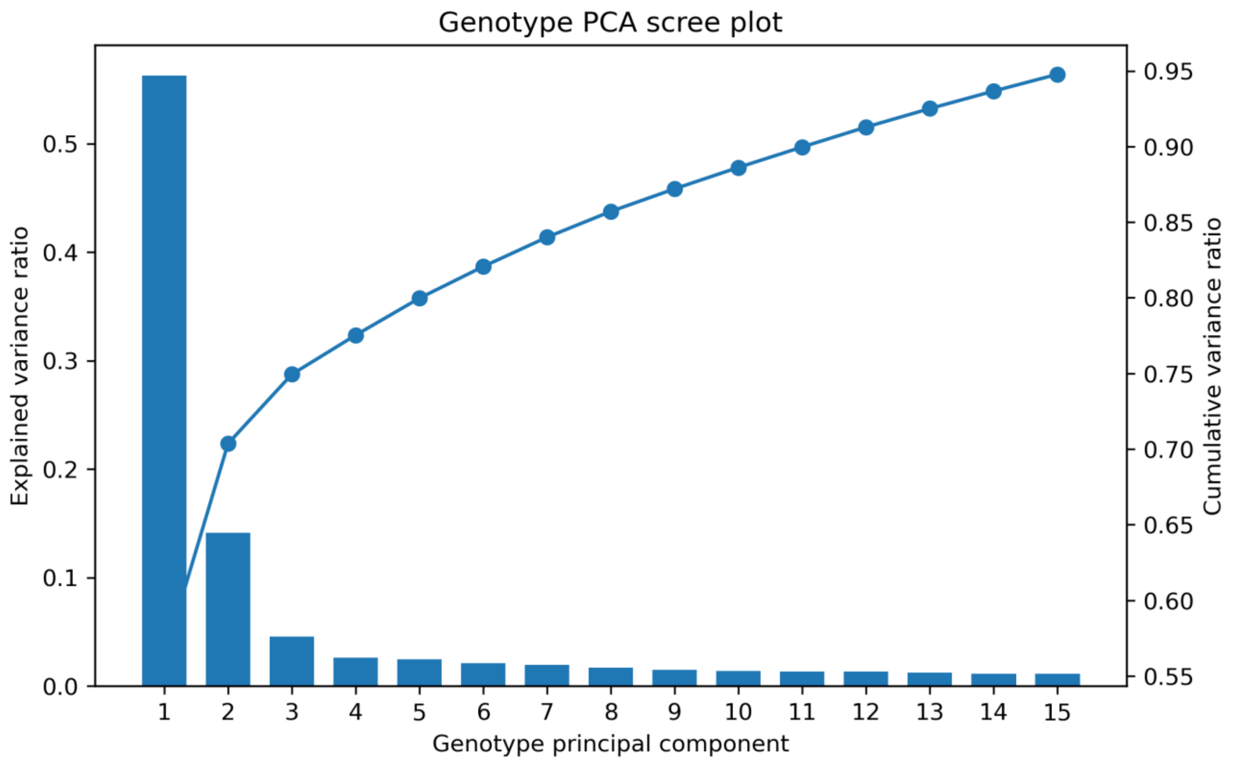

Supplementary Figure 7. Scree plot of genotype principal components used for population-structure summarization. Variance explained by the leading genotype PCs derived from the filtered SNP matrix. The first 10 PCs were retained for downstream analyses because they captured the major structure of the panel and accounted for most of the sample-space variance.

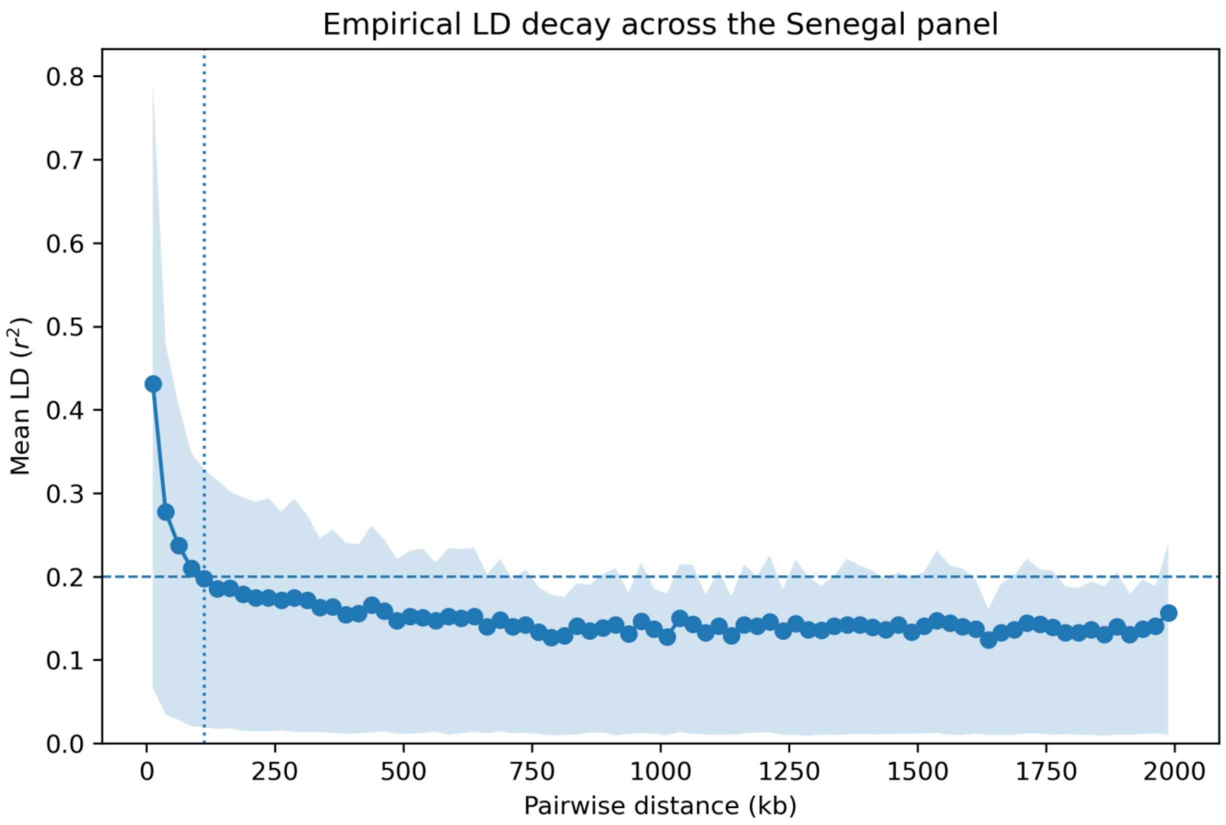

Supplementary Figure 8. Empirical linkage disequilibrium decay in the filtered sorghum SNP panel. Pairwise LD (r^2) was summarized as a function of physical distance using the filtered SNP set employed for the primary GWAS. The dashed horizontal line marks $r^2 = 0.2$, and the corresponding decay distance is indicated on the plot. The observed decay supported the use of a conservative ±250 kb operational window for grouping nearby association signals into QTL intervals during revision-stage interpretation.

Supplementary Data 1. Dataset overview and trait definitions. This file contains combined phenotypic data (main spectral traits and inter-seed uniformity metrics), significant SNPs, and candidate genes identified in this study.
